# Human papillomavirus infection can alter the level of tumour stemness and T cell infiltration in patients with head and neck squamous cell carcinoma

**DOI:** 10.3389/fimmu.2022.1013542

**Published:** 2022-11-07

**Authors:** Lingzhang Meng, Heming Lu, Yueyong Li, Jingjie Zhao, Siyuan He, Zechen Wang, Jiajia Shen, Huixian Huang, Jinru Xiao, Suren Rao Sooranna, Jian Song

**Affiliations:** ^1^ Institute of Cardiovascular Sciences, Guangxi Academy of Medical Sciences, Nanning, China; ^2^ Center for Systemic Inflammation Research (CSIR), School of Preclinical Medicine, Youjiang Medical University for Nationalities, Baise, China; ^3^ Second Division of Department of Radiation Oncology, Guangxi Academy of Medical Sciences, Nanning, China; ^4^ Second Division of Department of Radiation Oncology, the People’s Hospital of Guangxi Zhuang Autonomous Region, Nanning, China; ^5^ Department of Interventive Medicine, The Affiliated Hospital of Youjiang Medical University for Nationalities, Baise, China; ^6^ Life Science and Clinical Research Center, Affiliated Hospital of Youjiang Medical University for Nationalities, Baise, China; ^7^ Graduate School, Guangxi University of Chinese Medicine, Nanning, China; ^8^ Department of Metabolism, Digestion and Reproduction, Imperial College London, Chelsea & Westminster Hospital, London, United Kingdom

**Keywords:** human papillomavirus, single cell transcriptomic, head and neck squamous cell carcinoma, tumour stemness, tumour infiltrating immune cells, immune checkpoint genes, prognosis markers

## Abstract

Head and neck squamous cell carcinoma (HNSCC) usually has a poor prognosis and is associated with a high mortality rate. Its etiology is mainly the result from long-term exposure to either alcohol, tobacco or human papillomavirus (HPV) infection or a combination of these insults. However, HNSCC patients with HPV have been found to show a survival advantage over those without the virus, but the mechanism that confers this advantage is unclear. Due to the large number of HPV-independent HNSCC cases, there is a possibility that the difference in prognosis between HPV-positive (HPV^+^) and negative (HPV^-^) patients is due to different carcinogens. To clarify this, we used scRNA data and viral tracking methods in order to identify HPV^+^ and HPV^-^ cells in the tumour tissues of patients infected with HPV. By comparing HPV^+^ and HPV^-^ malignant cells, we found a higher level of tumour stemness in HPV^-^ tumour cells. Using tumour stemness-related genes, we established a six-gene prognostic signature that was used to divide the patients into low- and high-risk groups. It was found that HPV patients who were at low-risk of contracting HNSCC had a higher number of CD8^+^ T-cells as well as a higher expression of immune checkpoint molecules. Correspondingly, we found that HPV^+^ tumour cells expressed higher levels of CCL4, and these were highly correlated with CD8^+^ T cells infiltration and immune checkpoint molecules. These data suggest that the stemness features of tumour cells are not only associated with the prognostic risk, but that it could also affect the immune cell interactions and associated signalling pathways.

## Introduction

Head and neck squamous cell carcinoma (HNSCC) refers to cancers of the oral cavity, oropharynx and larynx and is the sixth most common cancer worldwide. HNSCC arises mainly as a result of exposure to carcinogens (e.g. alcohol and/or tobacco) or through malignant transformation due to human papillomavirus (HPV) infection. As most cases are locally advanced disease, HNSCC is associated with a poor prognosis, leading to high mortality rates. However, HPV-associated HNSCC appears to exhibit several unique biologically and clinically relevant features, with the presence of HPV conferring a survival advantage compared to its absence. A specific tumour-infiltrating immune population of CD8^+^ T cells was identified in HPV^+^ HNSCC patients, and this consisted of a greater proportion of dysfunctional cells (1).

HPV^+^ patients also appear to have a higher response rate to the programmed death 1 (PD-1) receptor and PD-1 ligand (PD-L1) ratio when compared to HPV^-^ patients (2). PD-1 and PD-L1 belong to a family of immune checkpoint proteins that act as co-inhibitory factors that can minimize the development of the T cell response. The receptor/ligand interactions ensure that the immune system is activated at the appropriate time in order to reduce the risk of chronic autoimmune inflammation (3). Blocking these immune checkpoint proteins by using monoclonal antibodies have been shown to aid the immune system to overcome a cancer’s ability to resist the immune response and stimulate the defence mechanisms against the cancer (4, 5). However, the underlying mechanisms and potential associations between the HPV status of a patient with HNSCC and the tumour immune environment remain unclear and needs to be further investigated.

Single-cell RNA sequencing (scRNA-seq) is an emerging technology that allows judgements to be made about individual cells and facilitates the study of highly heterogeneous cell populations (6). In virology, scRNA-seq studies not only allow the identification of different cell types, which interact with various pathogens, but also allow the accurate detection of virally expressed RNA at the single cell level, thus detecting pathogen-infected cells and distinguishing them from neighbouring bystander cells (1, 6).

In this study, we analysed the clinical data of HPV^+^ HNSCC patients and their scRNA-seq profiles. This allowed the comparison of HPV^+^ tumour cells with bystander tumour cells in order to investigate the relationships of HPV and the progression of HNSCC. We found that HPV^-^ tumour cells had a higher level of tumour stemness, which was closely associated with the poor prognosis of patients with HPV^-^ HNSCC. More importantly, we found that the enhanced tumour stemness scoring was correlated with a reduced number of infiltrated CD8 T cells in these HPV^-^ HNSCC patients. Our data also showed a significantly decreased CCL4 directed interaction of the immune cells in HPV– HNSCC patients compared to those who were HPV^+^. This might account for the degree of immune infiltration of cells and the efficiency of checkpoint therapy in HPV^+^ HNSCC patients.

## Materials and methods

### Cell culture

HPV^+^ (UM-SCC-47, UM-SCC-104) and HPV^-^ HNSCC (UM-SCC-6, UM-SCC-17A, UM-SCC-1) cell lines were purchased from Sigma. UPCI : SCC090 were from ATCC. Cells were incubated in DMEM medium supplemented with 1% L−glutamine, 10% FBS and 100 U/mL penicillin/streptomycin for 24h in an atmosphere of 5% CO_2_:95% air. The glioma cells in the co-culture system were collected and RNA was extracted by using Trizol. Transcriptome sequencing was performed by Novogene and differential gene expression analyses were performed by using the R software package, DESeq2, according to the manufacturer’s instructions.

### HNSCC RNAseq dataset

The bulk RNA sequencing and the clinical data were obtained from the HNSCC dataset of TCGA database, which consisted of 487 samples (https://portal.gdc.carcinoma.gov/). Each probe ID received an annotation with respect to the gene from the corresponding platform annotation profile of the GDC website and the raw matrix data received the quantile normalization and log2 conversion. As smoking is the independent risk factor for HNSCC, we excluded the patients who smoked. HPV groupings for HNSCC patients were based on the HPV condition which provided by the Pan-Cancer Microbiome (cBioportal.org). 72 of the 249 remaining patients were HPV^+^ and 177 were HPV^-^. The raw gene expression datasets were processed. Samples with missing data were excluded from this study.

### Correlation between HPV and survival of HNSCC patients

The information for the presence of HPV originated from the pan cancer dataset. These data were registered to the clinical data from TCGA accordingly (7). Correlations between the presence of HPV and the survival of HNSCC patients were studied as described in the following methods.

### ScRNA-seq analysis

The scRNA-seq data of patients with HNSCC were obtained from the GEO database (GSE164690) (8). The data were integrated using the SCTransform method of R software Seurat package and analysed using mutual principal component analysis (PCA). Clustering was performed in a resolution of the FindClusters that set to 0.1 and visualized with the uniform manifold approximation and projection (UMAP). Unique molecular modifier (UMI) counts of less than 500, doublets and cells with >5% of their mitochondrial genes were filtered out.

Viral mapping was performed as previously described (7). Raw scRNA fastq data were processed with UMI-tools (https://github.com/CGATOxford/UMI-tools).Virus infected cells were defined with the Viral-Track approach (6). In brief, the sequencing data containing the single cell index were mapped to the virus genome reference database and the status of the single cell was added to the expression matrix so as to correlate with the presence of HPV infection and the corresponding transcriptome.

### Tumour stemess analyses

For the cell line, we calculated mRNA expression-based stemness indices (mRNAsi) using one-class logistic regression machine-learning algorithm (OCLR). For the scRNA data, we employed the trajectory analysis using Monocle 2 package.

### Functional assay

GO ontology and KEGG pathway analyses were performed using the WebGestalt webserver. Cell- communication analyses was performed using the CellphoneDB R package (9). The Monocle2 R package was used for the trajectory analysis (10).

### HPV PCR

Primers were used to amplify the HPV 16 E6 region as follows: E6 forward primer, 5’-TCAGGACCCACAGGAGCG-3′, reverse primer, 5’-CCTCTCACGTCGCAGTAACTGTTG-3′. β-actin, which was used as housekeeping gene, was amplified by using forward primer 5’-TCACCCACACTGTGCCCATCTACGA-3′, reverse primer, 5’-CAGCGGAACCGCTCATTGCCAATGG-3′. HPV^+^ refers to the mean fluorescence intensity values above 1.5-fold negative control tissues.

### Immunohistochemistry

Immunofluorescence staining was performed as described previously (11). In brief, after embedding with a frozen section compound (Leica, #3801480), biopsies were sectioned into 4-μm on a microtome (Leica CM1950). For immunofluorescent staining, the sections were fixed in pre-cooled methanol (-20°C) for 5 minutes, after washing twice with PBS. The sections were then blocked with PBS/5%BSA/Fcγ blocker at 4°C for 1 hour. Primary antibodies were incubated with sections at 4°C overnight. After washing twice with PBS, the sections were incubated with fluorescent-coupled secondary antibodies for 1 hour at room temperature. After two further washes, the sections were mounted and imaged on an immunofluorescence microscope (Leica DMI3000B).

Antibodies used for staining included: Mouse-anti-human CD3 monoclonal antibody (Invitrogen, # MA1-21454), Rabbit-anti-human CTLA4 polyclonal antibody (Invitrogen, # PA5-115060), Rabbit-anti-human PD1 polyclonal antibody (Invitrogen, # PA5-20350), Rabbit-anti-human CCL4 polyclonal antibody (Invitrogen, #PA5-114961), Rabbit-anti-human FGFR2 polyclonal antibody (Invitrogen, #PA5-14651), A594 goat-anti-rabbit IgG (Affinity, #S0006), A488 donkey-anti-mouse IgG (Invitrogen, #A-21202) and DAPI (Invitrogen, #D21490).

### Statistical methods

Statistical studies were performed with the R software. Kaplan-Meier (KM) and receiver operating characteristic (ROC) tests were carried out as previously described (12–14), which was done with the “survivor” and “survROC” packages (15). The ‘survminer’ package (16) was used to calculate the optimal cut-off data points. We assessed the prognostic correlates of potentially interesting genes with univariate and multivariate Cox regression correlations. For prognostic correlates, we used the hazard ratios and 95% confidence intervals. Differences between groups were analysed using GraphPad Prism 8.0 software. Comparisons between the two groups were made using the Student’s t-test and P<0.05 was considered to be statistically significant.

## Results

### HPV infection correlates with low-risk HNSCC patients

**Figure 1:**
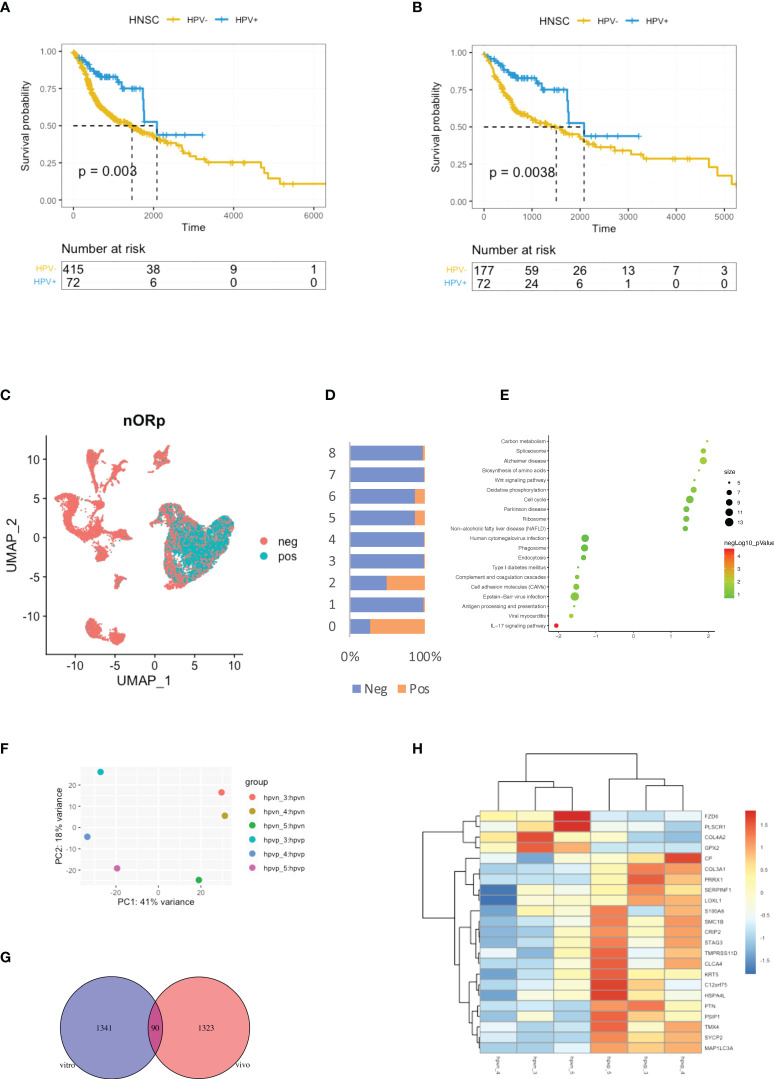
Virus analysis of the prognosis of high and low-risk groups of HNSCC patients. **(A)** Kaplan-Meier survival analysis of the HPV^+^ group in HNSCC patients. **(B)** Kaplan-Meier survival analysis of the HPV^+^ group in HNSCC patients who did not smoke. **(C)** UMAP diagrams showing HPV^+^ and HPV^-^ cells. **(D)** The bar graph quantifies and compares the proportion of HPV^+^ and HPV^-^ cells in the main cell types. **(E)** A bubble plot showing the enriched gene ontology of GSEA KEGG analysis of the DEGs in the HPV^+^ cluster 0 cells. **(F)** A dot plot showing the PCA analysis of the 3 HPV^+^ or HPV^-^ cell lines. **(G)** A Venn diagram showing the overlapping number of DEGs from the HPV^+^ tumour cells and HPV^-^ cell lines. **(H)** A heatmap showing the expression of the overlapped DEGs in the HPV^+^ and HPV^-^ cell lines.

HPV groupings for HNSCC patients were based on the HPV condition which provided by the Pan-Cancer Microbiome (cBioportal.org). Kaplan-Meier curves showed shorter survival times for the HPV^-^ patients when compared to those who were HPV^+^ (**Figure 1A**). As smoking is the independent risk factor for HNSCC, the patients who smoked were excluded so that 72 HPV^+^ and 177 HPV^-^ cases remained. The survival time was shorter in the HPV^-^ group compared to HPV^+^ patients after the smokers were excluded (**Figure 1B**). In order to be able to study the effect of HPV on HNSCC tumour cells, the scRNA-seq data were used to study HPV infection *in vivo*. During data mining, the HNSCC cells were divided into nine clusters, of which clusters 0 and 2 were tumour cells (**Figure 1C**, **sFigures 1A, B**). Approximately half of the cells in cluster 2 tumour cells were found to be infected with HPV, while more than 75% of cluster 0 tumour cells were infected (**Figure 1D**).

Differentially expressed genes (DEGs) in HPV^+^ and HPV^-^ tumour cells and GSEA functional analysis of these genes clearly indicated that HPV^-^ tumour cells upregulated the genes associated with the IL17 signalling pathway (**Figure 1E**, **sFigure 1B**). This correlates with increasing evidence that supports the pro-tumourigenic mechanisms of IL17 signalling in the immunosuppressive endogenous microenvironment within the growing tumour (17). To further investigate the intrinsic effects of HPV on tumour cells, three different types of HPV^+^ or HPV^-^ tumour cells were cultured. Transcriptomic analyses were performed on HPV^+^ and HPV^-^ tumour cells obtained from these experiments. PCA analysis revealed significant differences between HPV^+^ and HPV^-^ tumour cells (**Figure 1F**). Gene expression analysis identified 1341 DEGs between HPV^+^ and HPV^-^ tumour cells and 90 of these overlapped with the DEGs found between HPV^+^ and HPV^-^ cells *in vivo* (**Figure 1G**). A heatmap using these genes with a log fold change of more than 1 was constructed (**Figure 1H**). Although the presence of HPV in cells can lead to significant differences in gene expression, the data obtained from the cell lines and *in vivo* also show significant variations, suggesting the importance of the tumour microenvironment.

### Stemness of HPV-infected tumour cells

**Figure 2:**
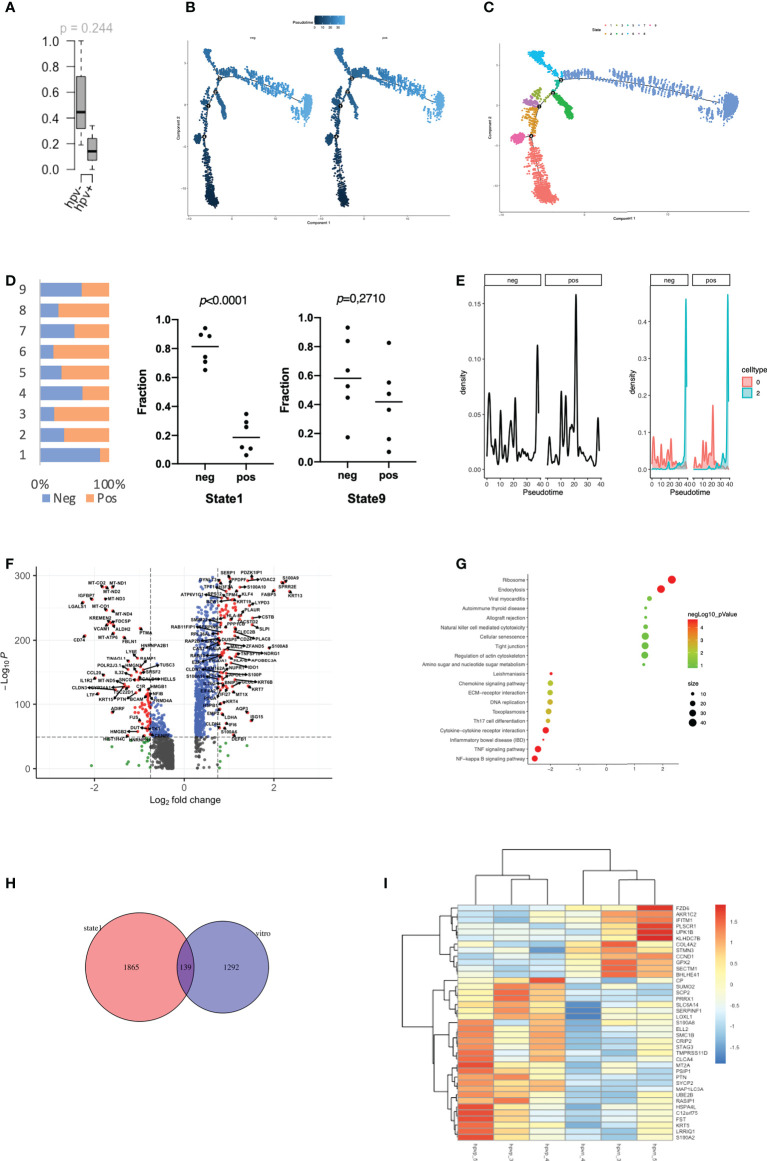
Stemness of HPV-infected tumour cells. **(A)** Boxplots to show the mRNAsi in the HPV^+^ or HPV^-^ cell lines (n=3). **(B)** Pseudotime of HPV^+^ and HPV^-^ tumour cells. **(C)** Trajectory analysis of HPV^+^ and HPV ^-^ tumour cells after identification of 9 states. **(D)** The bar chart indicates the ratio of HPV^+^ and HPV^-^ tumour cells in different trajectory states. **(E)** Ridge plots showing the distribution of HPV^+^ and HPV^-^ tumour cells on the pseudo-time. **(F)** A volcano plot showing the DEGs of state 1 tumour cells and their related functions. **(G)** A bubble plot showing the DEGs of state 1 tumour cells and their related functions. **(H)** A Venn diagram showing the overlapping number of DEGs from the state 1 tumour cells and the HPV^+^ cell lines. **(I)** A heatmap showing the expression of the overlapped DEGs in the HPV^+^ and HPV^-^ cell lines.

The stemness of tumour cells is closely related to the malignancy and recurrence of tumours (18). In the transcriptomic data from cell lines, we found a high level of stemness in HPV^-^ tumour cells, despite the lack of significance in the data due to the small sample size (**Figure 2A**). We therefore studied the level of stemness of tumour cells using scRNA data, in which we can identify genes that are in a transitional state between two different cell fates. Trajectory analysis was performed on HPV^+^ and HPV^-^ tumour cells, and we identified nine trajectory states in HNSCC tumour cells, with states 1 and 9 pointing to early stages of pseudo-time and therefore correlating with stemness of the tumour (**Figures 2B, C**). HPV^-^ tumour cells showed a high proportion of cancer stem cells (state1) compared to HPV^+^ cells (**Figure 2D**). While the expression seen in cluster 2 of both HPV^+^ and HPV^-^ tumour cells was upregulated in the later states, a higher proportion of HPV^+^ cells in the cluster 0 displayed a developed manner compared to the HPV^-^ cells (**Figure 2E**). This indicated an enhanced level of stemness in HPV^-^ tumour cells. DEGs of the early state (state1) showed a reduction in cytokine and chemokine signalling pathways (**Figures 2F, G**), while the HPV^+^ state 1 tumour cells showed a reduced level of IL17 signalling. This was similar to the DEGs of HPV^+^ cluster 0 cells (**Figure 1E**, **sFigures 2A, B**).

To further investigate the genes associated with stemness in the tumour cell lines, we compared the stemness-related genes that were differentially expressed in the HPV^+^ tumour cell lines cultured *in vitro* and found that 134 of them overlapped with the state 1 stemness genes (**Figure 2H**). A heatmap was constructed using the genes with a fold change of more than 1, in which we found that many DEGs in either HPV^+^ or HPV^-^ tumour cells remained in the same category (**Figure 2I**). Both *in vitro* and *in vivo* data implied a tendency of high stemness in the HPV^-^ tumour cells.

### The gene signature associated with stemness

**Figure 3:**
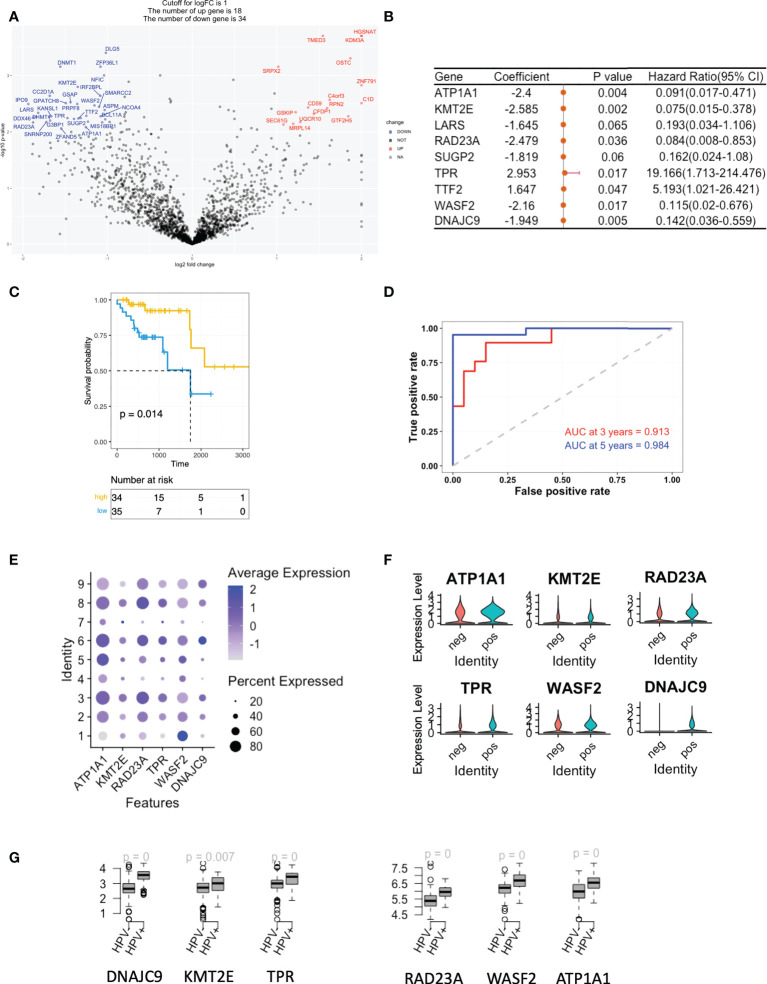
A gene signature based on the scoring of the level of stemness. **(A)** A volcano plot showing the stemness-related genes obtained from Cox regression analysis of survival-related HPV^+^ HNSCC patients. **(B)** Forest plot lines of the stemness-related top genes screened by using random survival forest analysis of HPV^+^ HNSCC patients. **(C)** Kaplan-Meier analysis of the risk groups that were defined with six gene tags associated with cell stemness in the TCGA dataset for HPV^+^ HNSCC. **(D)** Three- and five-year ROC survival curves of the risk groups from the TCGA dataset for HPV^+^ HNSCC. **(E)** Dot plots indicating the expression of the six signature genes in the nine states that were identified by trajectory analysis of HPV^+^ and HPV^-^ tumour cells. **(F)** Violin plots indicating the expression of the six signature genes in the HPV^+^ and HPV^-^ tumour cells. **(G)** Boxplots showing the the expression levels of the signature genes in the TCGA HPV^+^ and HPV^-^ patients. p=0 refers to p<0.0001.

As mentioned, we studied the level of stemness of HNSCC tumour cells using both cell lines and scRNA data. We filtered then the DEGs of the low stemness cells using the TCGA bulk-seq data. 34 risk negatively-related stemness genes were filtered for a univariate Cox regression study and these were found to significantly correlate with a reduced risk of HNSCC (p < 0.05; **Figure 3A**). By using the random survival forest algorithm, which is a powerful non-parametric method that can construct predictive models with time-to-event outcome, the top significant 6 genes were screened. These were ATP1A1, KMT2E, RAD23A, TRR, WASF2 and DNAJC9 (**Figure 3B**). The HNSCC patients in the TCGA dataset were then divided into groups with high- or low-risk, based on the expression levels of the gene signature. Kaplan–Meier curves showed that patients in the high-risk group had longer survival times than those in the low-risk group (**Figure 3C**). ROC curves obtained from the cases of HNSCC were plotted by estimating the predictive power of the genetic characteristics, and AUCs of 0.913 and 0.984 were obtained for 3- and 5-year survival rates, respectively (**Figure 3D**). The expression of the 6 signature genes were at a low level in state 1, but at a high level in the HPV^+^ group (**Figures 3E, F**). This was observed for both the scRNA and TCGA data (**Figures 3F, G**). Up to this point, we defined the stemness-based low and high risk TCGA samples using signature genes and compared the level of these signature genes in the HPV^+^ tumour cells and TCGA HNSCC cohort. Both data showed that the HPV situation is associated with the level of stemness-based signature genes.

We also performed a similar analysis on the positively-related stemness genes and found that the majority were correlated with an increased risk of HNSCC (**sFigures 3A, C**). 3 of these (ALDOA, CTNNA1 and TMBIM6) were significantly different, although the AUCs for the 3- and 5-year survival rates were only 0.705 and 0.76, respectively (**sFigures 3B, D**). These genes were highly expressed in state 1 but were similar in both the HPV^+^ and HPV – groups (**sFigures 3E, F**).

### The relationship between different HNSCC risk groups and immune checkpoint genes

**Figure 4:**
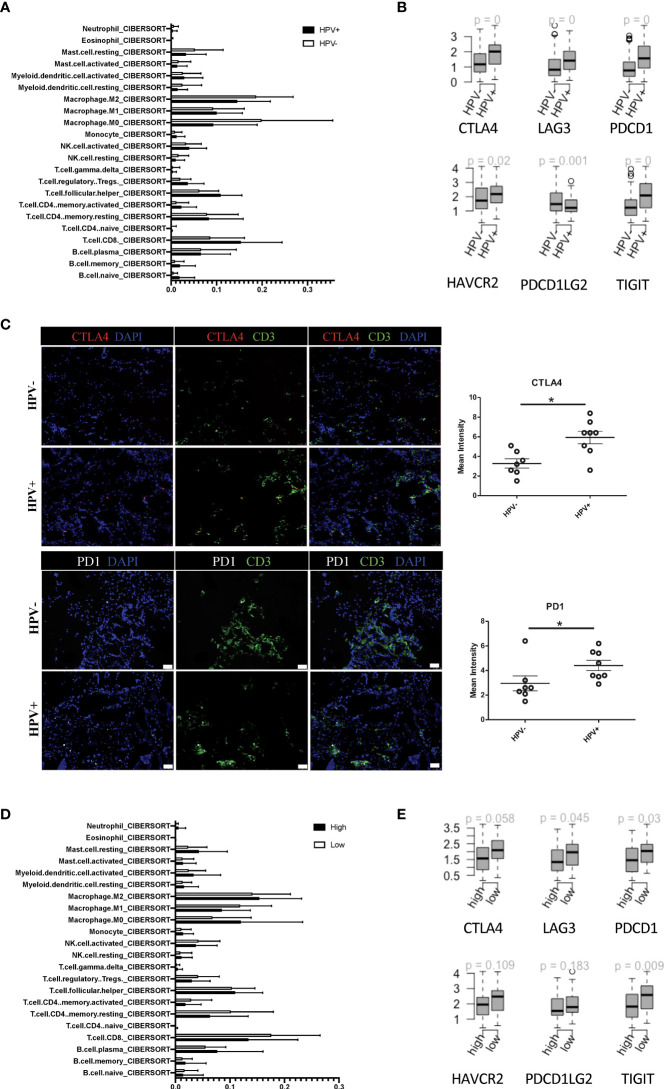
Tumour infiltrated immune cells in HPV^+^ and HPV^-^ HNSCC patients. **(A)** A bar graph showing the CIBERSORT estimated infiltration of immune cell subsets of samples from HPV^+^ and HPV^-^ HNSCC patients. **(B)** The relationship between HPV groups and immune checkpoint genes. Boxplots showing the gene expression levels of CTLA4, LAG3, PDCD1, PDCD1LG2, TIGIT and HAVCR2 in the HPV^+^ and HPV^-^ HNSCC patients. p=0 refers to p<0.0001. **(C)** Representative immuno-stained photomicrographs of CTLA4 and PD1 in surgical sections obtained from patients with HNSCC. The CD3 stain shows the tumour infiltrated T cells. The data are means ± SD of 7 experiments and were analysed using Student’s t test; *P<0.05. **(D)** A bar graph showing the CIBERSORT estimated infiltration of immune cell subsets in the high- and low-risk groups of HPV^+^ HNSCC patients. **(E)** Boxplots showing the gene expression levels of CTLA4, LAG3, PDCD1, PDCD1LG2, TIGIT and HAVCR2 in the high- and low-risk groups of HPV^+^ HNSCC patients.

In agreement with the previous studies, CIBERSORT estimated that the infiltration of immune cell subsets showed a greater proportion of CD8^+^ T cells in the HPV^+^ HNSCC patients when compared to those who were HPV^-^ (**Figure 4A**). Also, the immune checkpoint genes, including CTLA4, LAG3, PDCD1, HAVCR2, PDCD1LG2 and TIGIT, were found to be higher in the HPV^+^ HNSCC patients (**Figure 4B**). Similar results were also observed with the scRNA data (**sFigures 4A, B**). To confirm the checkpoint protein expression, we performed immunostaining on the surgical sections from the HPV^+^ and HPV^-^ HNSCC patients (**Figure 4C**). We observed significantly higher levels of CTLA4 and PD1 in the HPV^+^ HNSCC samples and a higher proportion of dysfunctional CD8^+^ T cells were found in HPV^+^ HNSCC patients (**Figure 4C**). Risk scores of HNSCC data and subtypes were evaluated according to the 6-gene signature that was established to be stemness-related genes. CIBERSORT estimated infiltration showed that the low-risk group of HNSCC patients had a higher proportion of CD8^+^ T cells (**Figure 4D**). In addition, the gene profiles showed that the immune checkpoint genes, CTLA4, LAG3, PDCD1 and TIGIT, were significantly increased in these patients (**Figure 4E**), suggesting a correlation between the stemness-related genes and the immune microenvironment.

### Cell communication between immune and HPV^-^infected tumour cells

**Figure 5:**
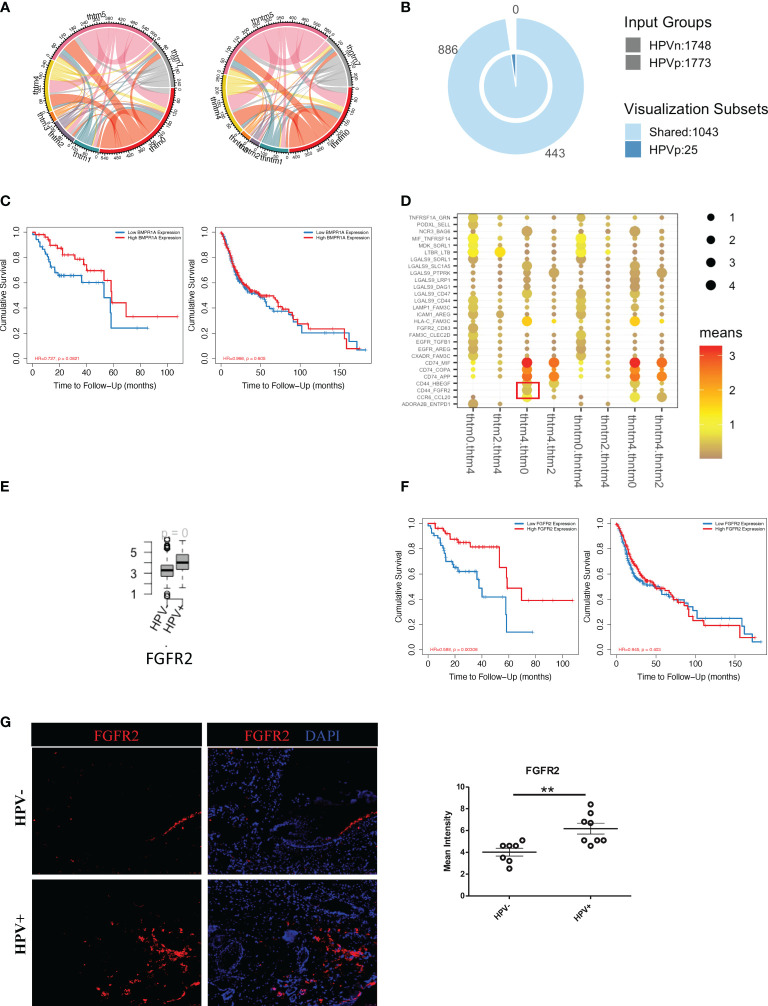
Cell communication between immune cells and HPV^+^ tumour cells. **(A)** A network of interactions between immune and either HPV^+^ or HPV^-^ tumour cells. The lines represent the pointing relationships. **(B)** A Venn diagram showing the shared and unique cell-cell interactions of immune cells with HPV^+^ or HPV^-^ tumour cells. **(C)** Kaplan-Meier survival analysis of BMPR1A in the HPV^+^ and HPV^-^ HNSCC patients. **(D)** Dot plots showing the most significant interactions of B cells with either HPV^+^ or HPV^-^ tumour cells and the significance of their relationships. The horizontal coordinates are cell-type interactions and the vertical coordinates are protein interactions, with the larger dots indicating smaller p-values and the colours representing the average expression. **(E)** Boxplots showing the gene expression of FGFR2 in the HPV^+^ and HPV^-^ HNSCC patients. p=0 refers to p<0.0001. **(F)** Kaplan-Meier survival analysis of FGFR2 in the HPV^+^ and HPV^-^ HNSCC patients. **(G)** Representative immuno-stained photomicrographs of FGFR2 in surgical sections obtained from patients with HPV^+^ and HPV^-^ HNSCC. The data are means ± SD of 7 experiments and were analysed using Student’s t test; **P<0.01.

To investigate the effect of HPV^+^ or HPV^-^ tumour cells on immune cells, we constructed an intercellular communication network and analysed the interactions between tumour cells and immune cells (**Figure 5A**). HPV^+^ tumour cells displayed 25 extra cell-cell interactions when compared to HPV^-^ cells, and of these BMPR1A showed the most promise as a favorable gene for the prognosis of HPV^+^ HNSCC patients. However, this was not the case for the HPV^-^ HNSCC patients (**Figures 5B, C**). When the B cells, T cells and macrophages interacted with tumour cells, it was found that interactions with the FGFR2 gene were upregulated, which was not detected in the endothelial cell-tumour interactions (**Figures 5D, E**, **sFigures 5A-C**). The FGFR2 gene could also be used as a possible candidate for the prognosis of HPV^+^ HNSCC (**Figure 5F**). To confirm the FGFR2 expression, we performed immunostaining on the surgical sections from the HPV^+^ and HPV^-^ HNSCC patients and observed a significantly higher level of FGFR2 in the samples of those who were positive (**Figure 5G**).

**Figure 6:**
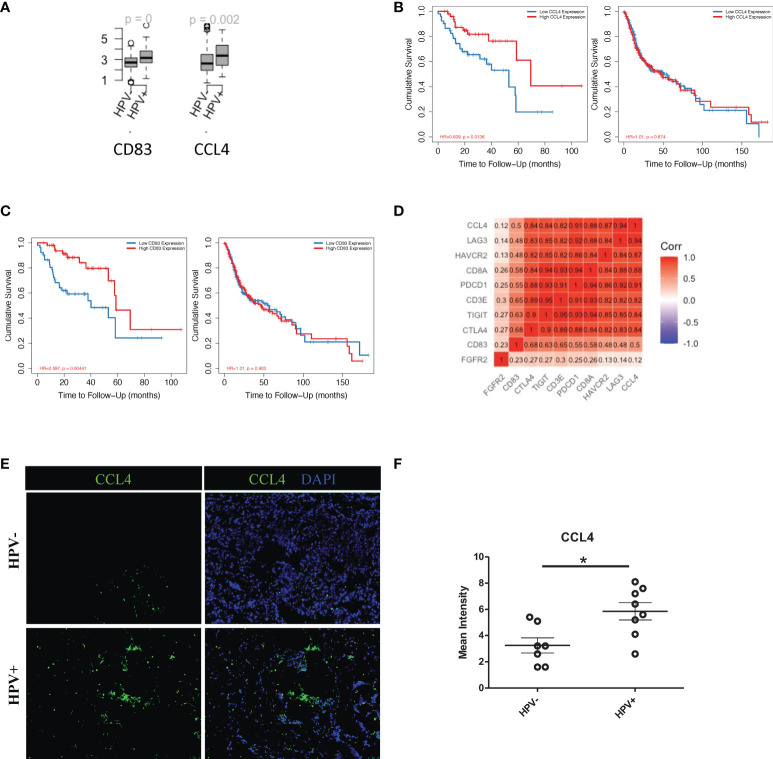
Cell communication between T cells and HPV^+^ tumour cells. **(A)** Boxplots showing the gene expression of CCL4, CD83 and FGFR2 in the HPV^+^ and HPV^-^ HNSCC patients. p=0 refers to p<0.0001. **(B)** Kaplan-Meier survival analysis of CCL4 in the HPV^+^ and HPV^-^ HNSCC patients. **(C)** Kaplan-Meier survival analysis of CD83 in the HPV^+^ and HPV^-^ HNSCC patients. **(D)** A correlation plot showing the correlations between the expression levels of CCL4 and CD83 with those of the checkpoint related genes. **(E)** Representative immuno-stained photomicrographs of CCL4 in surgical sections obtained from patients with HPV^+^ and HPV^-^ HNSCC. **(F)** Dot plot showing the quantification of immunostainings. The data are means ± SD of 7 experiments and were analysed using Student’s t test; *P<0.05.

More importantly, in this study, HPV^+^ tumour cells appeared to have a significantly higher level of cellular communication with CD8^+^ T cells, which correlated with the high proportion of tumour infiltrated CD8^+^ T cells in HPV^+^ HNSCC patients (**sFigure 5C**). The chemokine, CCL4, and the T cells activator, CD83, were also found to be upregulated in the HPV^+^ tumour-T cell interactions (**Figure 6A**). These were also genes that were upregulated in the HPV^+^ group and were significantly associated with a favorable prognosis in the HPV^+^ HNSCC patients (**Figures 6B, C**). Gene correlation assays showed that both CCL4 and CD83 not only correlated with the amount of recruited T cells, but also had a substantial correlation with the immune checkpoint genes such as CTLA4, LAG3, PDCD1, HAVCR2 and TIGIT (**Figure 6D**). Furthermore, the expression of CCL4 was validated and quantified using immunostaining, and this showed a significantly higher level of CCL4 in the HPV^+^ HNSCC samples (**Figure 6E, F**). The data indicated an important role of the interactions between HPV^+^ tumour cells and infiltrated T cells, which might account for the different prognoses observed in HPV^+^ and HPV^-^ HNSCC patients.

## Discussion

**Figure 7:**
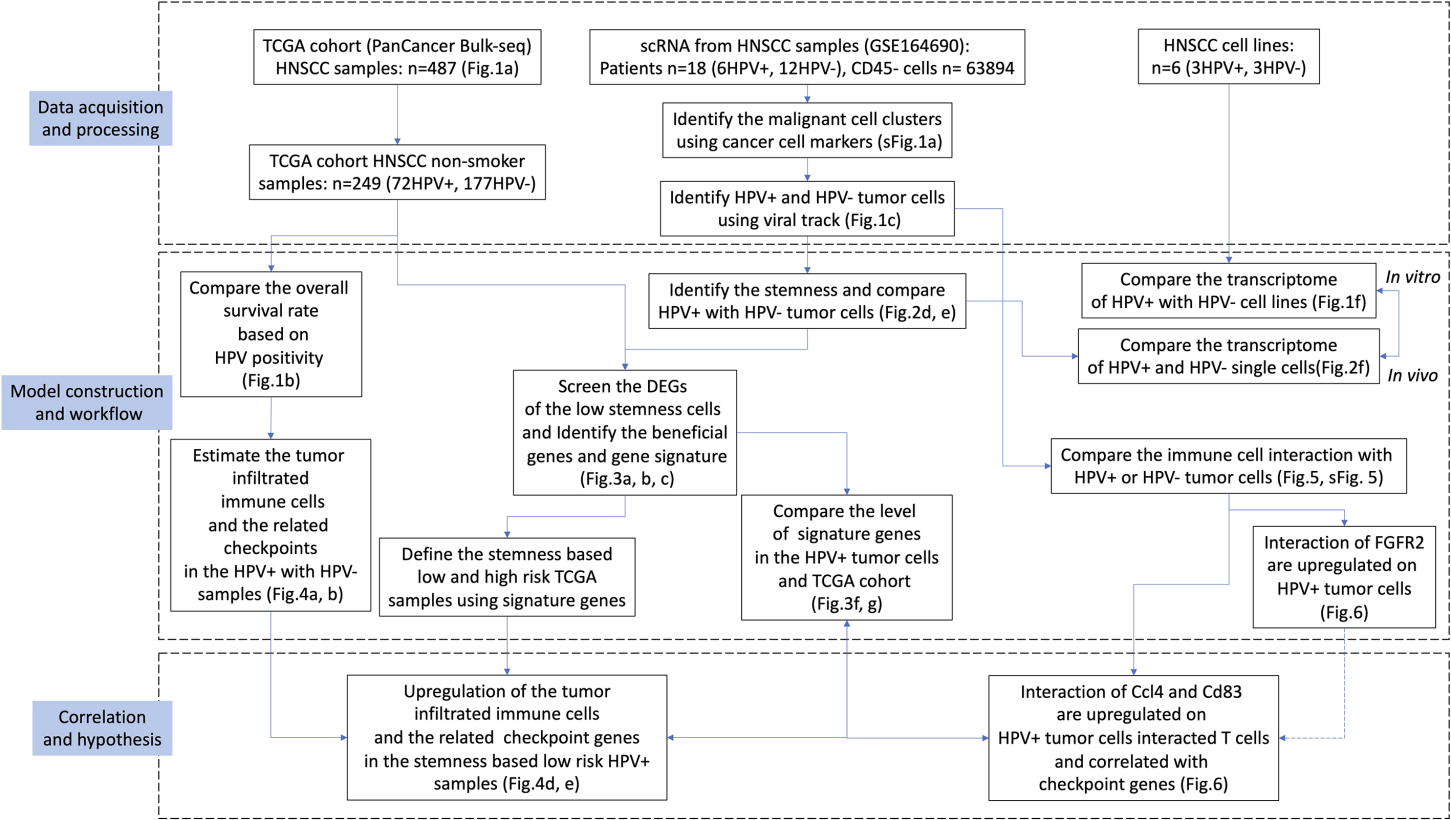
Workflow of our study.

HNSCC is a type of cancer that undergoes malignant transformation as a result of exposure to carcinogens (e.g. alcohol and/or tobacco) or as a result of HPV infection. HPV-associated HNSCC has been found to exhibit unique biologically and clinically relevant features, and patients with the virus show a survival advantage when compared to those without. However, due to the large number of HPV-independent HNSCC, there is the possibility that the prognostic differences seen between HPV^+^ and HPV^-^ cases are due to their different etiologies as well as the pathogenesis of the disease. In order to clarify this, we used scRNA data and viral tracking methods to identify HPV^+^ and HPV^-^ cells in tumour tissues obtained from HPV-infected patients with HNSCC. A tumour stemness analysis for HPV-associated DEGs was performed and it was found that HPV^-^ tumour cells had a higher level of tumour stemness (**Figure 7**).

In this study, we are only exploring the reasons why patients with HPV^+^ tumours have a better prognosis compared to HPV^-^ patients. The association of HPV with a better prognosis does not necessarily mean that HPV is not involved in the development of cancer. It is possible that negative cases were infected at an earlier time point before cancer diagnosis, and the fact that HPV negativity is usually associated with advanced tumours, suggests that these viruses may become undetectable at a later stage of the carcinogenic process (19). Our data revealed a high proportion of tumour cells contain HPV, and we therefore hypothesized that HPV-containing cells in tumour tissue are likely to lose their internal mutational control during the oncogenic process. This would allow these cells to subsequently acquire the potential for gene expression and cancer cell recurrence that are associated with malignant mutations. In contrast, in the case of HPV posivity, tumour cells may be better controlled by the immune system due to the expression of viral proteins, leading to a relatively more promising prognosis for these patients.

As mentioned, we studied the level of stemness of HNSCC tumour cells using scRNA data. We filtered then the DEGs of the low stemness cells using the TCGA bulk-seq data. These top significant 6 genes, including ATP1A1, KMT2E, RAD23A, TRR, WASF2 and DNAJC9, were screened from the 34 negatively-related stemness genes, using the random survival forest algorithm. ATP1A1, KMT2E and WASF2 were known as the hallmarker of cancers and overexpression of these genes promote the survival of cancer cells (20–22), while RAD23A acts as a negative regulator of anti-virus response and might correlate directly with the HPV infection (23). TPR is a nucleoporin which prevents DNA damage (24) and DNAJC9 is a dual histone chaperone and heat shock cochaperone, which recruits HSP70 enzymes to guard histone structural integrity (25). Both TPR and DNAJC9 might have dual function in term of tumour biology. Although it is hard to understand how the gene signature was constructed based on the known function of each gene, the signature genes are very specific for the HPV^+^ group in term of both the expression and the prognostic risk.

Our data here reveals that HPV^+^ patients at low risk of HNSCC had higher numbers of CD8^+^ T cells and a higher expression level of immune checkpoint genes. We found that HPV^+^ tumour cells expressed higher levels of CCL4 by analysis of their cell-to-cell interactions and that this expression was highly correlated with CD8^+^ T cells and immune checkpoint molecules. CCL4 is known to enable the active recruitment of CD8^+^ cytotoxic T lymphocytes (26), which in turn leads to the diffusion of the chemokines, CCL3 and CCL4, and accelerate the recruitment of distant T cells through long-distance homotypic signalling (27). Our data here re-emphasises the importance of CCL4 in the regulation of the immune microenvironment of HPV^+^ HNSCC.

In the current study, clinical and scRNA-seq data from HNSCC patients were analysed to explore the relationship and mechanisms of high-risk HPV and the progression of HNSCC. This led us to determine the degree of immune infiltration, and the signalling pathways associated with HPV infection, with a view to revealing potential therapeutic targets associated with HNSCC. Our data suggested that HPV^-^ HNSCC cells contained more properties associated with cancer stemness, both in terms of analysis and experimental validation of the gene expression profiles in tumour cell lines incubated *in vitro*. The level of stemness is not only associated with prognostic risk of tumours, but it can also affect the immune cell interactions and some signalling pathways associated with HPV^+^ HNSCC. Our study here revealed several novel potential targets for therapeutic options for patients with HNSCC. In addition, it may provide a potential mechanism for the development of a specific therapy regimen to combat both HPV^+^ and HPV^-^ HNSCC. The limitation of current work is the limited number of patients that included in the scRNA analyes. Also, the multi-scaling method that integrates the bulk-seq data and scRNA might lead to certain bias due to the different number of factors involved.

The treatment of recurrent or metastatic HNSCC has long been similar. Recent advances in immunotherapy have given patients and physicians the option of omitting chemotherapy. In a small percentage of patients, immunotherapy has induced durable disease control. However, it remains unclear which patients can be prioritised for immunotherapy. Our current research has the potential to help HNSCC take one more step towards more personalised medicines.

## Data availability statement

The original contributions presented in the study are included in the article/**Supplementary Material**. Further inquiries can be directed to the corresponding author.

## Ethics statement

The studies involving human participants were reviewed and approved by Ethics Committee of Youjiang Medical University for Nationalities. The patients/participants provided their written informed consent to participate in this study.

## Author contributions

JS designed this study. LM, HL, YL and JZ performed analysis of the scRNA-seq data. SH, ZW and JJS performed the bulk sequencing & immunofluorescent staining. HH and JX helped to collect the data for this study. SS helped to compose and revise the manuscript. All authors contributed to the article and approved the submitted version.

## Funding

This study was funded by grants from the National Science Foundation of China (#31970745), Guangxi Natural Science Foundation (#2020GXNSFAA259050) and Youjiang Medical University for Nationalities (#yy2019bsky001) and Research Program of The Affiliated Hospital of Youjiang Medical University for Nationalities (#R20212601).

## Conflict of interest

The authors declare that the research was conducted in the absence of any commercial or financial relationships that could be construed as a potential conflict of interest.

## Publisher’s note

All claims expressed in this article are solely those of the authors and do not necessarily represent those of their affiliated organizations, or those of the publisher, the editors and the reviewers. Any product that may be evaluated in this article, or claim that may be made by its manufacturer, is not guaranteed or endorsed by the publisher.

## Supplementary material

Supplementary Figure 1 ScRNA analysis of the HPV^+^ and HPV^-^ HNSCC-infiltrated cells. **(A)** Violin plots indicating the expression and distribution of marker genes. cluster 1 and 2 refer to tumor cells; cluster 1 T cells; cluster 3 plasma cells; cluster 4 and 8 B cells; cluster 5 endothelial cells; cluster 6 stroma cells; cluster 7 macrophages. **(B)** UMAP diagrams showing the clusters of the HNSCC-infiltrated cells. **(C)** Volcano plots demonstrating the expression patterns and levels of the genes in HPV^+^ HNSCC tumour cells.

Supplementary Figure 2 Stemness of HPV-infected tumour cells. A volcano plot **(A)** and bubble plot **(B)** showing the DEGs of the HPV-infected state 1 tumour cells.

Supplementary Figure 3 Gene signatures based on the stemness scoring of cells. **(A)** A volcano plot showing the stemness-related genes obtained from Cox regression analysis of survival-related HPV^-^ HNSCC patients. **(B)** Forest plot lines of the stemness-related three top genes screened by using random survival forest analysis of HPV^-^ HNSCC patients. **(C)** Kaplan-Meier analysis of the risk groups that were defined with three gene tags in the TCGA dataset for HPV^-^ HNSCC patients. **(D)** Three- and five-year ROC survival curves of the risk groups from the TCGA dataset for HPV^-^ HNSCC patients. **(E)** Dot plots indicating the expression of three signature genes in the nine states that were identified by trajectory analysis of HPV^+^ and HPV^-^ tumour cells. **(F)** Violin plots indicating the expression of three signature genes in the HPV^+^ and HPV^-^ tumour cells.

Supplementary Figure 4 Tumour-infiltrated immune cells in HPV^+^ and HPV^-^ HNSCC tumour cells. **(A)** A bar graph showing the immune cell subsets obtained from the scRNA data from HPV^+^ and HPV^-^ HNSCC patients. **(B)** Violin plots showing the expression levels of CTLA4, LAG3, PDCD1, PDCD1LG2, TIGIT and HAVCR2 in the T cells.

Supplementary Figure 5 Cell-cell communication between immune cells and HPV-infected tumour cells. Dot plots showing the most significant interactions (mean>1) of **(A)** endothelial cells, **(B)** macrophages and **(C)** T cells with either HPV^+^ or HPV^-^ tumour cells and the significance of their relationships. The horizontal coordinates are cell-type interactions and the vertical coordinates are protein interactions, with the larger dots indicating smaller p-values and the colours representing the average expression.

## Abbreviations

HNSCC, Head and neck squamous cell carcinoma; HPV, Human papillomavirus; scRNA-seq, Single-cell RNA sequencing; UMAP, Uniform manifold approximation and projection; UMI, Unique molecular modifier; DEGs, Differentially expressed genes; KM, Kaplan-Meier; ROC, Receiver operating characteristic; mRNAsi, mRNA expression-based stemness indices; OCLR, One-class logistic regression machine-learning algorithm.
