# Supplementary figures and images for: Human papillomavirus infection can alter the level of tumour stemness and T cell infiltration in patients with head and neck squamous cell carcinoma
Source: Front Immunol. 2022 Nov 7;13:1013542. doi: 10.3389/fimmu.2022.1013542 (PMC9676257; doi:10.3389/fimmu.2022.1013542)

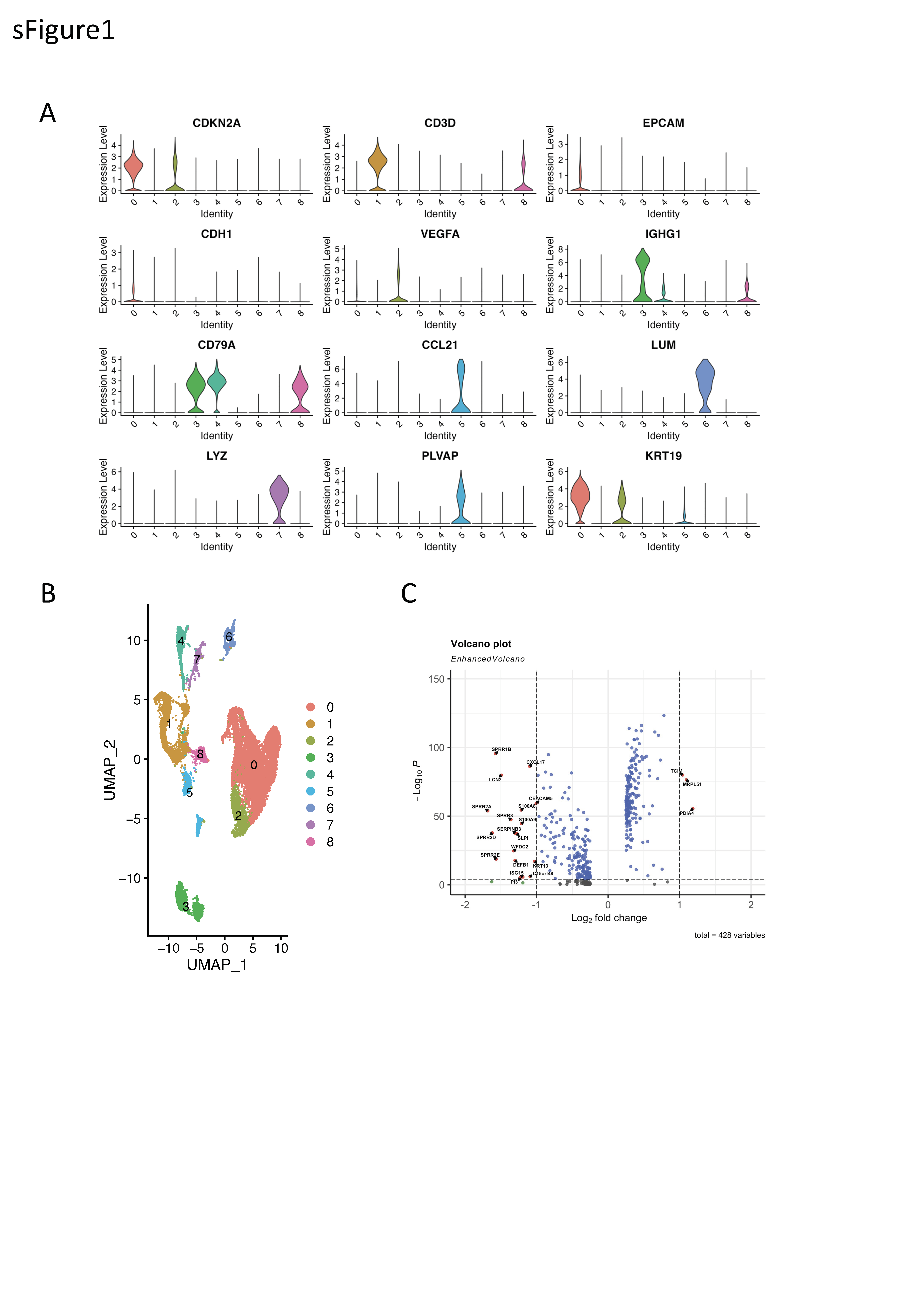

Supplement: Supplementary Figure 1 — ScRNA analysis of the HPV+ and HPV- HNSCC-infiltrated cells. (A) Violin plots indicating the expression and distribution of marker genes. cluster 1 and 2 refer to tumor cells; cluster 1 T cells; cluster 3 plasma cells; cluster 4 and 8 B cells; cluster 5 endothelial cells; cluster 6 stroma cells; cluster 7 macrophages. (B) UMAP diagrams showing the clusters of the HNSCC-infiltrated cells. (C) Volcano plots demonstrating the expression patterns and levels of the genes in HPV+ HNSCC tumour cells. [file Image_1.tiff]

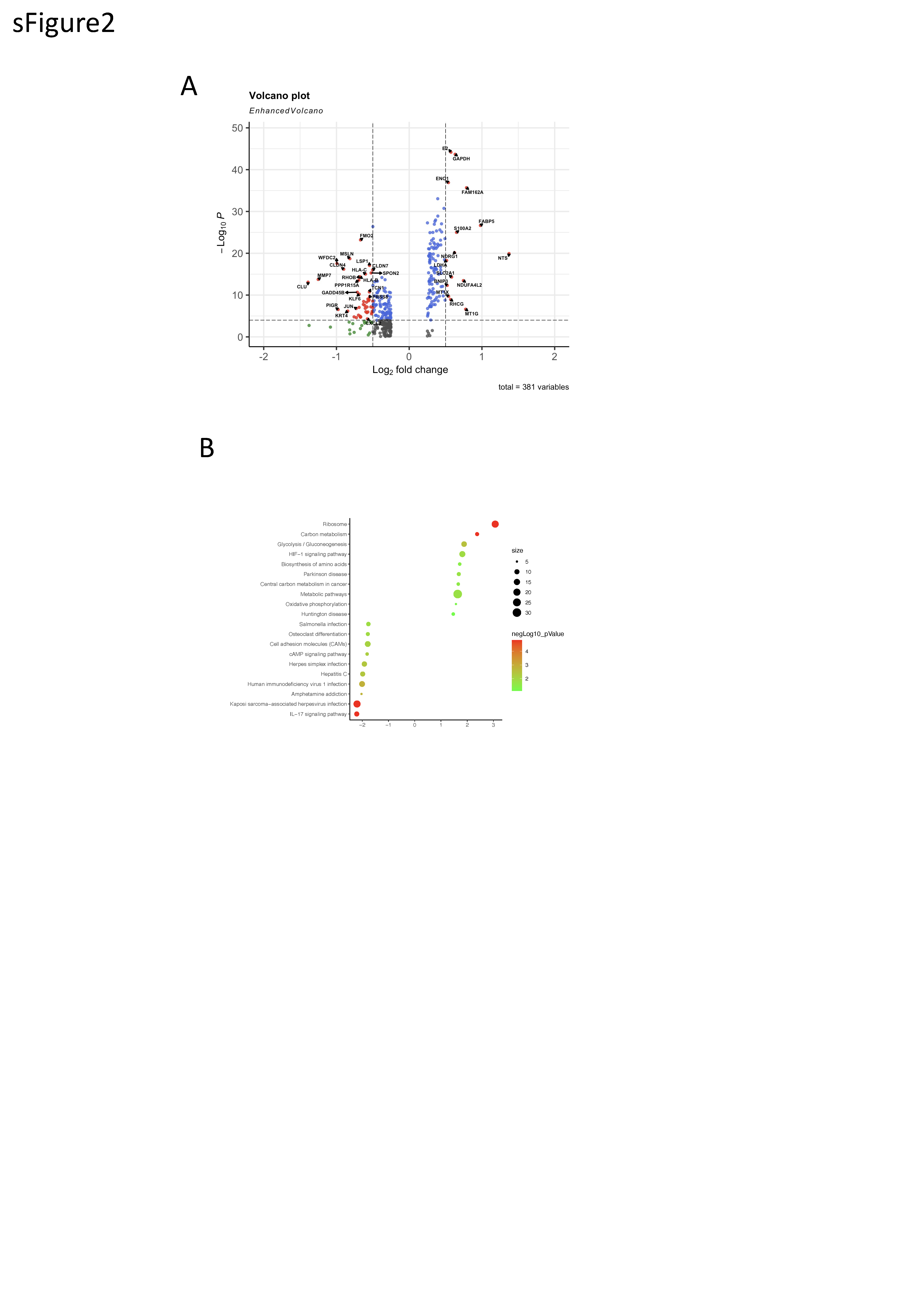

Supplement: Supplementary Figure 2 — Stemness of HPV-infected tumour cells. A volcano plot (A) and bubble plot (B) showing the DEGs of the HPV-infected state 1 tumour cells. [file Image_2.tiff]

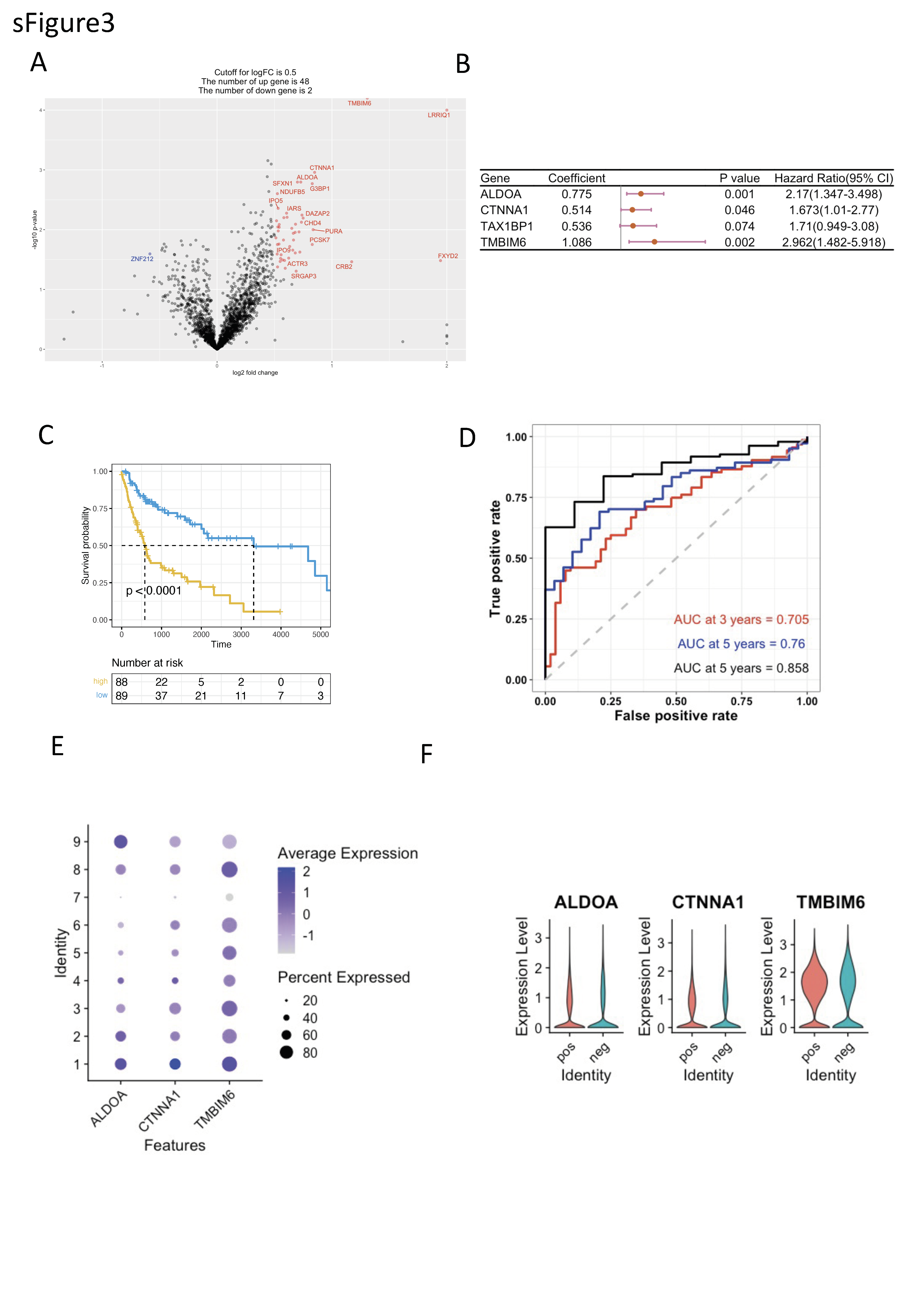

Supplement: Supplementary Figure 3 — Gene signatures based on the stemness scoring of cells. (A) A volcano plot showing the stemness-related genes obtained from Cox regression analysis of survival-related HPV- HNSCC patients. (B) Forest plot lines of the stemness-related three top genes screened by using random survival forest analysis of HPV- HNSCC patients. (C) Kaplan-Meier analysis of the risk groups that were defined with three gene tags in the TCGA dataset for HPV- HNSCC patients. (D) Three- and five-year ROC survival curves of the risk groups from the TCGA dataset for HPV- HNSCC patients. (E) Dot plots indicating the expression of three signature genes in the nine states that were identified by trajectory analysis of HPV+ and HPV- tumour cells. (F) Violin plots indicating the expression of three signature genes in the HPV+ and HPV- tumour cells. [file Image_3.tiff]

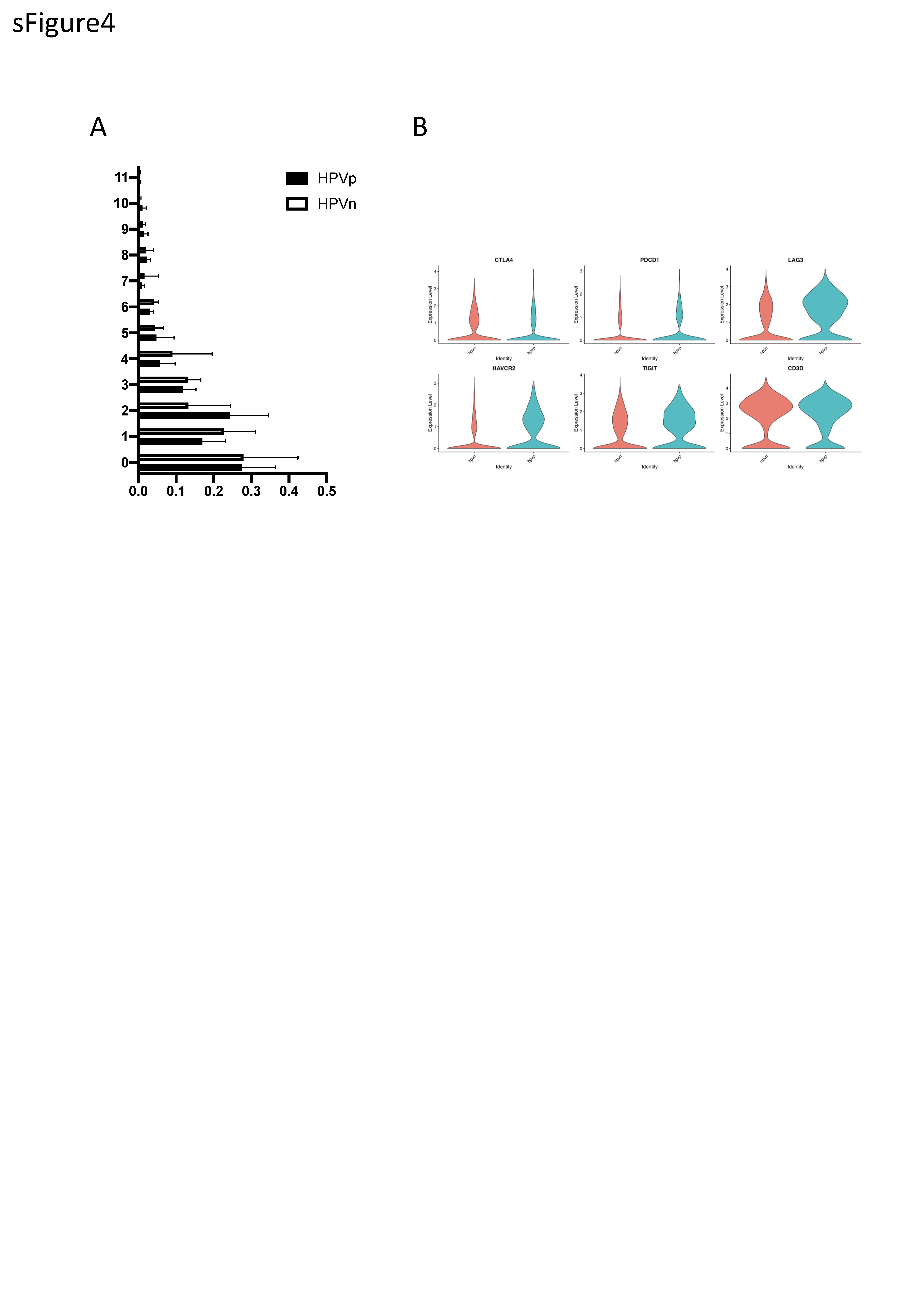

Supplement: Supplementary Figure 4 — Tumour-infiltrated immune cells in HPV+ and HPV- HNSCC tumour cells. (A) A bar graph showing the immune cell subsets obtained from the scRNA data from HPV+ and HPV- HNSCC patients. (B) Violin plots showing the expression levels of CTLA4, LAG3, PDCD1, PDCD1LG2, TIGIT and HAVCR2 in the T cells. [file Image_4.tiff]

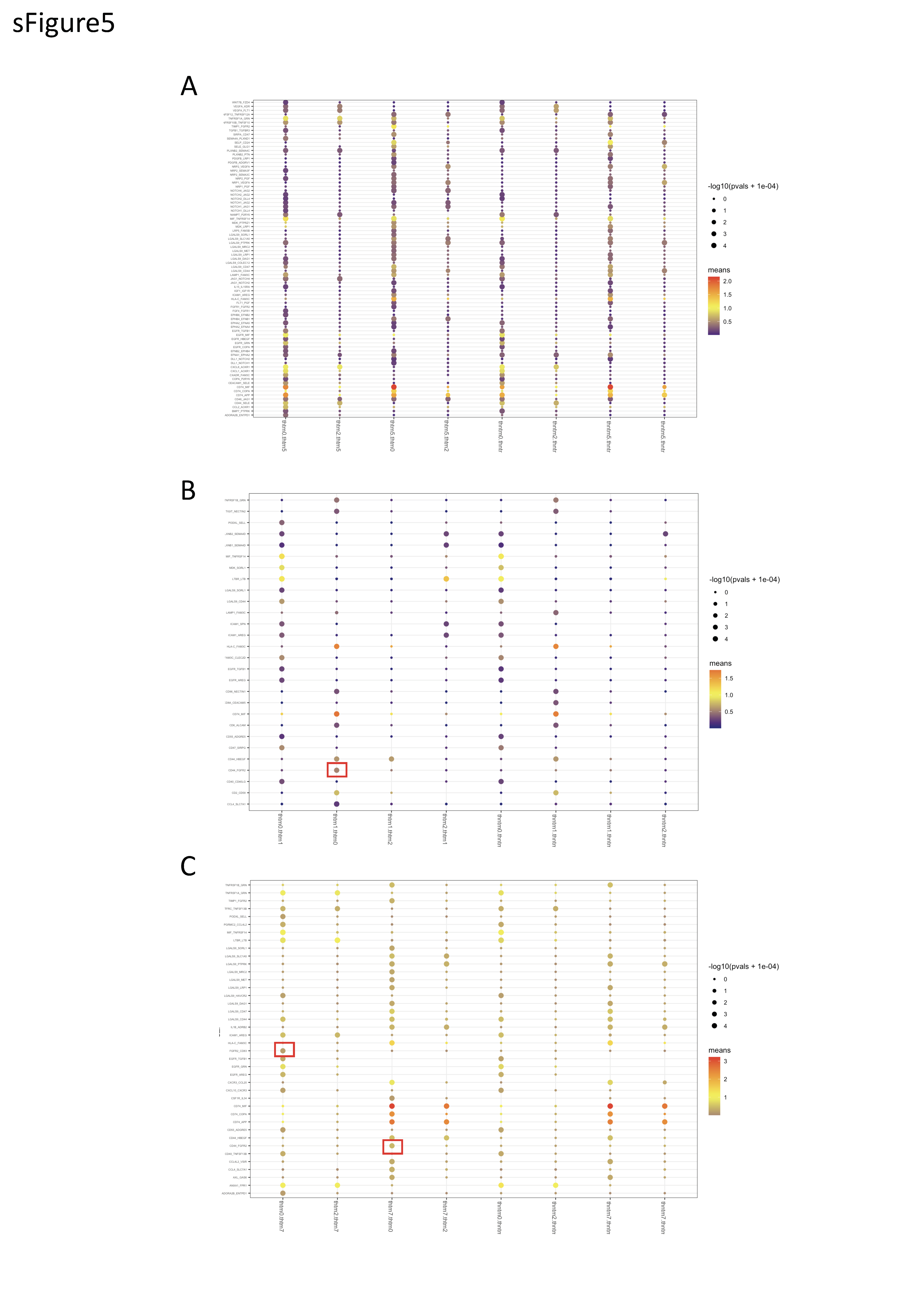

Supplement: Supplementary Figure 5 — Cell-cell communication between immune cells and HPV-infected tumour cells. Dot plots showing the most significant interactions (mean>1) of (A) endothelial cells, (B) macrophages and (C) T cells with either HPV+ or HPV- tumour cells and the significance of their relationships. The horizontal coordinates are cell-type interactions and the vertical coordinates are protein interactions, with the larger dots indicating smaller p-values and the colours representing the average expression. [file Image_5.tiff]
